# Association of Olfactory and Pulmonary Function in Middle-Aged and Older Adults: The Korea National Health and Nutrition Examination Survey

**DOI:** 10.3390/jcm10071535

**Published:** 2021-04-06

**Authors:** Ji-Sun Kim, Jun-Ook Park, Dong-Hyun Lee, Ki-Hong Chang, Byung Guk Kim

**Affiliations:** Department of Otolaryngology-Head and Neck Surgery, Eunpyeong St. Mary’s Hospital, College of Medicine, The Catholic University of Korea, Seoul 07345, Korea; skswltjs23@hanmail.net (J.-S.K.); junook2000@naver.com (J.-O.P.); cjidea@naver.com (D.-H.L.); khchang@catholic.ac.kr (K.-H.C.)

**Keywords:** pulmonary function, olfactory function, risk factor, obstructive, KNHANES

## Abstract

Objectives: To identify the relationship between pulmonary function and subjective olfactory dysfunction in middle-aged and older adults. Materials and Methods: We used Korea National Health and Nutrition Examination Survey data from 2010 to 2012 to analyze 6191 participants in their 50s or older. Results: The frequency of olfactory dysfunction was 6.8% among the subjects with normal pulmonary function tests, but was significantly more frequent in those diagnosed with restrictive (9.6%) or obstructive (10.1%) pulmonary function. Forced volume vital capacity, forced expiratory volume (FEV)_1_, FEV_6_, and peak expiratory flow were significantly lower in the olfactory dysfunction group. The risk of olfactory dysfunction was significantly associated with obstructive pulmonary function (odds ratio (OR) [95% confidence interval (CI)]: 1.449 [1.010–2.081]) after adjusting for confounders (sex, rhinitis, chronic rhinosinusitis, hypertension, dyslipidemia, education level, stress, depressed mood, and suicidal ideation). Conclusion: Middle-aged and older adults with obstructive pulmonary function had a higher incidence of subjective olfactory dysfunction than the normal pulmonary function group. Early olfactory testing may improve the quality of life of patients with obstructive pulmonary function.

## 1. Introduction

Olfactory dysfunction is a common upper respiratory symptom and has been reported to have a high prevalence of about 19% in the adult population [1]. Olfactory dysfunction affects diet and quality of life (QOL) and, in severe cases, can adversely affect mental health [2]. In addition, patients with olfactory dysfunction are exposed to the risk of accidents during daily life, such as inability to smell burning or gas, or eating rotten food [3]. Several questionnaire studies have confirmed that olfactory dysfunction has a significant impact on QOL [4]. As the aging society progresses, the prevalence of olfactory dysfunction is expected to increase further. Moreover, there are increasing individual demands to improve the QOL by improving the olfactory function. It is important to understand the causes and mechanisms of olfactory dysfunction for the treatment of it.

The causes of olfactory dysfunction are various including sinonasal disease, upper respiratory infection, trauma, chemical damage, old age, endocrine metabolic abnormalities, neurodegenerative diseases, tumors, congenital abnormalities, and iatrogenic causes [3]. Several population-based studies have reported on the risk factors of olfactory dysfunction. One of the most important risk factors for olfactory dysfunction is age, and it has been reported that older age has a higher incidence of olfactory dysfunction [5,6]. This is related to changes in the olfactory epithelium and a decrease in synaptic cells that transmit olfactory signals to the cerebrum that occur with age [7,8]. Therefore, the present study intended to identify risk factors for olfactory dysfunction in middle and old age, considering the age group with a high prevalence of olfactory dysfunction.

In this study, the Korea National Health and Nutrition Examination Survey (KNHANES), a cross-sectional survey conducted by the Korea Centers for Disease Control and Prevention (KCDCP), was used. Risk factors for olfactory dysfunction in middle and old age were identified, and in particular, the relationship between pulmonary function and olfactory dysfunction was confirmed.

## 2. Materials and Methods

### 2.1. Study Population

We used KNHANES data from 2010 to 2012. The survey included questionnaires and interviews about medical history, health-related behavior, and nutritional status. Each individual also undergoes a physical examination and blood sampling by medical staff. The total number of subjects calculated by variance estimation of the composite sample was 24,173, of which 9734 were in their 50s or older. The participants missing data for the parameters we wanted to study were excluded. Ultimately, 6191 participants were included in our analysis.

### 2.2. Ethical Considerations

All participants provided informed consent at baseline. The survey protocol was approved by the institutional review board of the KCDCP (IRB No. 2010-02CON-21-C, 2011-02CON-06-C, and 201201EXP-01-2C).

### 2.3. Survey of Olfactory Dysfunction

To assess olfactory function, participants were asked if they had problems with smelling sensation that lasted more than 3 months. Participants who responded positively and negatively were evaluated as having olfactory dysfunction and normal olfactory function, respectively.

### 2.4. Pulmonary Function Test

Trained technicians performed pulmonary function testing (PFT) on participants using a spirometry system (Model 2130; SensorMedics, CA, USA). Spirometry was repeated at least three times. The test was based on the technical standards for performing spirometry, jointly adopted by the American Thoracic Society and European Respiratory Society in 2005 [9]. Forced vital capacity (FVC), forced expiratory volume in 1 (FEV_1_) and 6 (FEV_6_) seconds, forced expiratory flow at 25–75% of FVC (FEF25–75%), and peak expiratory flow (PEF) were recorded. This study used FEV_1_, FVC, and the FEV_1_ to FVC ratio (FEV_1_/FVC). Predicted values were calculated using the Korean reference equations based on representative Koreans. In the spirometry findings, an obstructive pattern was defined as FEV_1_/FVC <0.70 and a restrictive pattern as FEV_1_/FVC ≥0.70 and FVC <80% predicted. FEV_1_/FVC ≥0.70 and FVC ≥80% were defined as normal pulmonary function.

### 2.5. Assessing Anthropometric and Laboratory Measurements

Medical staff measured the participants’ weight and height. Body mass index (BMI) was calculated as weight (kg)/height (m^2^). Underweight (BMI < 18.5 kg/m^2^) and obesity (BMI ≥ 25 kg/m^2^) were defined using the BMI cut-offs for Asians of the Regional Office for the Western Pacific Region of the World Health Organization [10]. Blood samples were taken after fasting 10–12 h overnight, and laboratory results were measured in serum.

### 2.6. Assessment of Health-Related Behaviors

Health-related behaviors were surveyed using a self-report questionnaire. Participants were asked whether they currently smoked or exercised regularly (defined as at least 20 min of intense physical activity three or more times a week). Mental health status was assessed by asking whether the participants ordinarily perceived moderate or severe stress, whether they had experienced feelings of depression for 2 weeks or more within the last year, and whether they had suicidal ideation within the last year.

### 2.7. Statistical Analysis

SPSS ver. 21 (SPSS, Chicago, IL, USA) was used for the analyses. To estimate the characteristics of people living in South Korea accurately, complex sample analysis was performed using the weight variables of KNHANES, reflecting strata and cluster. These sampling weights were used in all our analyses. The chi-square test was used to compare the characteristics between the normal and olfactory dysfunction groups. The independent *t*-test was used to compare the results of laboratory tests and PFT. Adjusted odds ratios (ORs) and the 95% confidence interval (CI) were calculated using multiple logistic regression analysis to investigate the association between pulmonary function patterns and olfactory dysfunction. We adjusted for age and sex (model 1) and then for these variables plus rhinitis, chronic rhinosinusitis, hypertension (HTN), and dyslipidemia (model 2), and finally adjusted model 2 for education level, stress, depressed mood, and suicidal ideation (model 3). *p* < 0.05 was considered statistically significant.

## 3. Results

### 3.1. Prevalence of Olfactory Dysfunction

Table 1 shows the characteristics of the study participants according to olfactory dysfunction. The overall prevalence of olfactory impairment was 7.8% (7.2% for men, 8.3% for women). The number of participants complaining of olfactory dysfunction increased significantly with age. The frequency of olfactory dysfunction among the subjects with normal PFT results was 6.8%, and was significantly higher in those diagnosed with restrictive (9.6%) or obstructive (10.1%) pulmonary function. In the chi-square analysis, BMI, HTN, diabetes, dyslipidemia, and anemia did not affect the prevalence of olfactory dysfunction. Subjects with rhinitis and chronic rhinosinusitis had a higher incidence of olfactory dysfunction, while there was no difference in the incidence of olfactory dysfunction according to current smoking and regular exercise. The frequency of olfactory dysfunction was significantly higher when subjects had low education levels or mental problems, such as high-stress perception, depressed mood, and suicidal ideation.

### 3.2. Differences in Laboratory and PFT Results According to Olfactory Dysfunction

Table 2 shows the differences in the laboratory and PFT results for the groups according to olfactory dysfunction. Diastolic, but not systolic blood pressure was significantly (independent *t*-test) lower with olfactory dysfunction. Serum total cholesterol and triglyceride were lower in the olfactory dysfunction group. Laboratory values related to anemia did not differ between the two groups. FVC, FEV_1_, FEV_6_, and PEF were significantly lower in the olfactory dysfunction group.

### 3.3. Associations between Olfactory Dysfunction and Pulmonary Function

Table 3 shows the association of the PFT results with the prevalence of olfactory dysfunction after adjusting for confounders. The adjusted OR for olfactory dysfunction was not significant with restrictive pulmonary function, but was significantly associated with obstructive pulmonary function after adjusting for confounders (OR 1.449, 95% CI 1.010–2.081 in Model 3).

## 4. Discussion

This study identified factors related to olfactory dysfunction using national epidemiological data. The main finding was that, even after adjusting for factors known to be associated with olfactory dysfunction, the group with an obstruction pattern on PFT had about 1.4 times higher olfactory dysfunction than the normal pulmonary function group. To the best of our knowledge, this is the first population-based study to confirm the association between pulmonary function and olfactory dysfunction.

A study that analyzed risk factors for olfactory dysfunction in adults over 20 years of age using KNHANES data in 2009 found that, in addition to older age, low-income, a history of hepatitis B, rhinitis, and chronic rhinosinusitis were associated with a high incidence of olfactory dysfunction [11]. Other KNHANES studies reported that low education and psychiatric factors such as high-stress perception, depression, and suicidal ideation were related to the occurrence of olfactory dysfunction [12,13,14]. It is difficult to compare these results with ours directly because the age groups and data collection years differ among the studies. However, our findings that rhinitis, chronic rhinosinusitis, education level, and psychiatric factors were risk factors for olfactory dysfunction were similar to previous findings.

Few studies have reported on the relationship between obstructive lung diseases or lung transplantation and olfactory function. One study reported that nasal polyps worsened owing to persistent asthma, leading to worsening olfactory function [15]. However, this shows that asthmatics are susceptible to developing nasal polyps rather than explaining the association between asthma and olfactory function. In a study that reported a relationship between chronic obstructive pulmonary disease (COPD) and olfactory function, there was a significant decrease in olfactory function in the COPD group compared with the healthy group [16]. In that study, olfactory function was not related to the presence of oxygen therapy in the COPD group, and there was no difference in the results of the olfactory tests between the COPD and healthy groups after adjusting for smoking. Another study found that the OR of anosmia in patients with stable COPD increased by 1.19% per year in outpatient care [17]. This means that COPD patients need a multidisciplinary approach to olfactory dysfunction, which may be related to their QOL. Patients waiting for lung transplantation and those who received a lung transplant were reported to have poor olfactory function [18]. Although lung transplantation did not improve olfactory function, olfactory function was an important factor in their QOL.

The olfactory nerve is the only nerve in the central nervous system that is exposed to the external environment and it is vulnerable to upper airway infection [19]. Patients with obstructive pulmonary disease have reduced mucociliary function compared with healthy individuals, so inflammation of the upper respiratory mucosa may easily occur [20]. Biochemical studies have reported increased inflammatory markers in the nasal mucosa of COPD patients [21]. Obstructive pulmonary disease can be diagnosed only if it is consistent with the patient’s symptoms, along with obstructive pulmonary function; we cannot say that all subjects with obstructive pulmonary function in this study had obstructive pulmonary disease. However, we can suggest that airway infections, which may occur frequently in middle and older age with obstructive pulmonary function, could be the cause of olfactory dysfunction. It has also been reported that olfactory function is significantly reduced in obstructive sleep apnea [22,23]. Those studies postulated that the problem with the olfactory nerve was due to intermittent hypoxia caused by obstructive sleep apnea. Similarly, one could postulate that olfactory impairment is more frequent in the group with pulmonary dysfunction because they are more susceptible to intermittent hypoxia than the normal group. Our analysis found that the incidence of olfactory dysfunction was higher in the restricted pulmonary function group than in the normal group, although the difference was not significant (9.6% vs. 6.8%, respectively). We believe that there may be a relationship between pulmonary and olfactory function, which causes intermittent hypoxia, although the difference in the incidence of olfactory dysfunction between restrictive and obstructive pulmonary function cannot be explained.

One limitation of this study was that olfactory function was not tested, although previous studies reported high correlations between subjective olfactory impairment and the results of psychophysical olfactory tests [24,25]. Future studies should investigate the relationship between olfactory and pulmonary function using psychophysical olfactory tests. In addition, this study used the PFT data for middle-aged and older adults only. The results for younger subjects could verify our assumptions about the correlation between pulmonary and olfactory function at all ages. As this was a cross-sectional study, the causal relationship between olfactory and pulmonary function cannot be determined.

This is the first study to report a relationship between pulmonary function and olfactory dysfunction. Even after adjusting for nasal factors such as rhinitis and chronic rhinosinusitis, pulmonary function was significantly related to the occurrence of olfactory dysfunction. This is important as it provides evidence that screening tests for olfactory function are needed to improve the QOL of patients with pulmonary dysfunction. This should be the basis for clinical research on therapeutic effects on olfactory dysfunction in patients with abnormal pulmonary function. Further laboratory and clinical studies should elucidate the pathophysiology of olfactory dysfunction related to our results.

## 5. Conclusions

In middle-aged and older adults, those with obstructive pulmonary function had an approximately 1.4 times higher incidence of olfactory dysfunction than the normal pulmonary function group. To improve the QOL of patients with pulmonary dysfunction, screening tests and treatment for olfactory dysfunction may be needed. Further clinical studies of the relationship between olfactory and pulmonary function are needed.

## Figures and Tables

**Table 1:** Prevalence of olfactory dysfunction according to participant characteristics.

	Olfactory Dysfunction Unweighted Count (Weighted %)		Total Unweighted Count	*p*-Value
	Yes	No		
**Age group** (**years**)				0.014 *
50–59	158 (6.2%)	2585 (93.8%)	2743	
60–69	174 (8.8%)	1976 (91.2%)	2150	
70–79	100 (8.9%)	1097 (91.1%)	1197	
≥80	13 (16.1%)	88 (83.9%)	101	
**Sex**				0.268
Men	178 (7.2%)	2469 (92.8%)	2647	
Women	267 (8.3%)	3277 (91.7%)	3544	
**PFT results**				0.021 *
Normal	294 (6.8%)	4111 (93.2%)	4405	
Restrictive	59 (9.6%)	633 (90.4%)	692	
Obstructive	92 (10.1%)	1002 (89.9%)	1094	
**Body mass index**				0.214
Underweight	8 (14.4%)	71 (85.6%)	79	
Normal	263 (7.4%)	3546 (92.6%)	3809	
Obesity	174 (8.1%)	2129 (91.9%)	2303	
**Hypertension**				0.432
No	262 (7.5%)	3616 (92.5%)	3878	
Yes	183 (8.2%)	2130 (91.8%)	2313	
**Diabetes**				0.270
No	381 (7.6%)	5034 (92.4%)	5415	
Yes	64 (9.0%)	712 (91.0%)	776	
**Dyslipidemia**				0.300
Yes	353 (7.6%)	4749 (92.4%)	5102	
No	92 (8.8%)	997 (91.2%)	1089	
**Anemia**				0.760
No	415 (7.8%)	5349 (92.2%)	5764	
Yes	30 (7.3%)	397 (92.7%)	427	
**Rhinitis**				
No	265 (5.6%)	4676 (94.4%)	4941	<0.0001 *
Yes	180 (16.5%)	1070 (83.5%)	1250	
**Chronic rhinosinusitis**				<0.0001 *
No	307 (5.8%)	5494 (94.2%)	5801	
Yes	138 (36.7%)	252 (63.3%)	390	
**Smoking**				0.358
Current smoker	66 (7.4%)	880 (92.6%)	946	
Former smoker	99 (6.4%)	1331 (93.6%)	1430	
Non-smoker	280 (7.9%)	3535 (92.1%)	3815	
**Education**				0.009 *
Low (< high school)	291 (8.7%)	3290 (91.3%)	3581	
High (≥ high school)	154 (6.4%)	2456 (93.6%)	2610	
**Regular exercise**				0.167
No	395 (8.0%)	4991 (92.0%)	5386	
Yes	50 (6.2%)	755 (93.8%)	805	
**Stress—Moderate to severe**				0.009 *
No	328 (7.1%)	4580 (92.9%)	4908	
Yes	117 (10.1%)	1166 (89.9%)	1283	
**Depressed mood**				0.021 *
No	357 (7.3%)	4967 (92.7%)	5324	
Yes	88 (10.4%)	779 (89.6%)	867	
**Suicidal ideation**				<0.0001 *
No	348 (7.0%)	4963 (93.0%)	5311	
Yes	97 (12.1%)	783 (87.9%)	880	

PFT: pulmonary function test. * Significant at *p* < 0.05.

**Table 2:** Comparison of the laboratory results according to olfactory dysfunction.

	Olfactory Dysfunction Weighted		*p*-Value
	Yes	No	
Systolic BP (mmHg)	125.04 ± 0.95	126.69 ± 0.33	0.089
Diastolic BP (mmHg)	75.86 ± 0.54	78.27 ± 0.20	<0.0001 *
Total cholesterol (mM)	189.49 ± 2.26	196.65 ± 0.65	0.002 *
Triglyceride (mM)	147.01 ± 5.43	147.65 ± 1.96	<0.0001 *
Hemoglobin (g/dL)	13.93 ± 0.08	14.09 ± 0.03	0.066
Hematocrit (%)	41.54 ± 0.23	41.84 ± 0.07	0.199
Ferritin (nM)	100.38 ± 7.50	97.84 ± 2.45	0.745
Serum Iron (uM)	111.10 ± 2.47	114.40 ± 0.75	0.200
TIBC (uM)	310.93 ± 2.83	312.21 ± 0.78	0.652
Spirometry			
FVC (L)	3.23 ± 0.06	3.36 ± 0.01	0.027 *
FVC (percent predicted, %)	91.06 ± 0.83	92.17 ± 0.20	0.193
FEV1 (L)	2.40 ± 0.04	2.53 ± 0.01	0.001 *
FEV1 (percent predicted, %)	91.25 ± 0.89	92.05 ± 0.25	0.381
FEV1/FVC	0.75 ± 0.01	0.76 ± 0.00	0.121
FEV6 (L)	3.10 ± 0.05	3.23 ± 0.01	0.010 *
FEF25–75% (L/sec)	3.24 ± 0.14	3.46 ± 0.08	0.082
PEF (L/sec)	6.48 ± 0.11	6.81 ± 0.04	0.004 *

Values are the weighted mean ± SE or % ± SE. BP: blood pressure; TIBC: total iron binding capacity; FVC: forced vital capacity; FEV: forced expiratory volume; FEF: forced expiratory flow; PEF: peak expiratory flow. * Significant at *p* < 0.05.

**Table 3:** Adjusted odds ratios of olfactory dysfunction according to pulmonary function.

PFT Patterns	Odds Ratio (95% Confidence Intervals)		
	Model 1	Model 2	Model 3
Normal	1	1	1
Restrictive	1.399 (0.889–2.203)	1.428 (0.886–2.300)	1.412 (0.875–2.278)
Obstructive	1.462 (1.042–2.051) *	1.448 (1.005–2.088) *	1.449 (1.010–2.081) *

Model 1 was adjusted for age and sex. Model 2 was adjusted for age, sex, rhinitis, chronic rhinosinusitis, HTN, and dyslipidemia. Model 3 was adjusted for age, sex, rhinitis, chronic rhinosinusitis, HTN, dyslipidemia, education level, stress, depressed mood, and suicidal ideation. * Significant at *p* < 0.05; PFT: pulmonary function test; HTN: hypertension.

## Data Availability

The dataset analyzed for this study can be found at https://knhanes.cdc.go.kr/knhanes/eng/index.do (accessed on 6 April 2021)

## References

[1] Brämerson A., Johansson L., Ek L., Nordin S., Bende M. (2004). Prevalence of olfactory dysfunction: The skövde population-based study. Laryngoscope.

[2] Croy I., Nordin S., Hummel T. (2014). Olfactory Disorders and Quality of Life—An Updated Review. Chem. Senses.

[3] Temmel A.F.P., Quint C., Schickinger-Fischer B., Klimek L., Stoller E., Hummel T. (2002). Characteristics of Olfactory Disorders in Relation to Major Causes of Olfactory Loss. Arch. Otolaryngol. Head Neck Surg..

[4] Toller S.V. (1999). Assessing the impact of anosmia: Review of a questionnaire’s findings. Chem Sens..

[5] Hoffman H.J., Ishii E.K., Macturk R.H. (1998). Age-Related Changes in the Prevalence of Smell/Taste Problems among the United States Adult Population: Results of the 1994 Disability Supplement to the National Health Interview Survey (NHIS). Ann. New York Acad. Sci..

[6] LaFreniere D., Mann N. (2009). Anosmia: Loss of Smell in the Elderly. Otolaryngol. Clin. North. Am..

[7] Rawson N.E., Gomez G., Cowart B., Restrepo D. (1998). The use of olfactory receptor neurons (ORNs) from biopsies to study changes in aging and neurodegenerative diseases. Ann. N. Y. Acad. Sci..

[8] Bhatnagar K.P., Kennedy R.C., Baron G., Greenberg R.A. (1987). Number of mitral cells and the bulb volume in the aging human olfactory bulb: A quantitative morphological study. Anat. Rec. Adv. Integr. Anat. Evol. Biol..

[9] Pellegrino R., Viegi G., Brusasco V., Crapo R.O., Burgos F., Casaburi R., Coates A., Van Der Grinten C.P.M., Gustafsson P., Hankinson J. (2005). Interpretative strategies for lung function tests. Eur. Respir. J..

[10] World Health Organization (2000). The Asia-Pacific Perspective: Redefining Obesity and Its Treatment.

[11] Lee W.H., Wee J.H., Kim D.-K., Rhee C.-S., Lee C.H., Ahn S., Lee J.H., Cho Y.-S., Lee K.H., Kim K.S. (2013). Prevalence of Subjective Olfactory Dysfunction and Its Risk Factors: Korean National Health and Nutrition Examination Survey. PLoS ONE.

[12] Kong I.G., Kim S.Y., Kim M.S., Park B., Kim J.H., Choi H.G. (2016). Olfactory Dysfunction Is Associated with the Intake of Mac-ronutrients in Korean Adults. PLoS ONE.

[13] Park D.-Y., Kim H.J., Kim C.-H., Lee J.Y., Han K., Choi J.H. (2018). Prevalence and relationship of olfactory dysfunction and tinnitus among middle- and old-aged population in Korea. PLoS ONE.

[14] Hwang S.-H., Kang J.-M., Seo J.-H., Han K.-D., Joo Y.-H. (2016). Gender Difference in the Epidemiological Association between Metabolic Syndrome and Olfactory Dysfunction: The Korea National Health and Nutrition Examination Survey. PLoS ONE.

[15] Alobid I., Cardelus S., Benítez P., Guilemany J.M., Roca-Ferrer J., Picado C., Bernal-Sprekelsen M., Mullol J. (2011). Persistent asthma has an accumulative impact on the loss of smell in patients with nasal polyposis. Rhinol. J..

[16] Dewan N.A., Bell C.W., Moore J., Anderson B., Kirchain W., O’Donohue W.J. (1990). Smell and Taste Function in Subjects with Chronic Obstructive Pulmonary Disease. Chest.

[17] Huerta A., Donaldson G.C., Singh R., Mackay A.J., Allinson J.P., Brill S.E., Kowlessar B., Torres A., Wedzicha J.A. (2015). Upper Respiratory Symptoms Worsen over Time and Relate to Clinical Phenotype in Chronic Obstructive Pulmonary Disease. Ann. Am. Thorac. Soc..

[18] Irani S., Thomasius M., Schmid-Mahler C., Holzmann D., Goetzmann L., Speich R., Boehler A. (2010). Olfactory performance before and after lung transplantation: Quantitative assessment and impact on quality of life. J. Heart Lung Transplant..

[19] Van Riel D., Verdijk R., Kuiken T. (2015). The olfactory nerve: A shortcut for influenza and other viral diseases into the central nervous system. J. Pathol..

[20] Yaghi A., Dolovich M.B. (2016). Airway Epithelial Cell Cilia and Obstructive Lung Disease. Cells.

[21] Hurst J.R. (2009). Upper airway. 3: Sinonasal involvement in chronic obstructive pulmonary disease. Thorax.

[22] Invitto S., Calcagnì A., Piraino G., Ciccarese V., Balconi M., De Tommaso M., Toraldo D.M. (2019). Obstructive sleep apnea syndrome and olfactory perception: An OERP study. Respir. Physiol. Neurobiol..

[23] Iannella G., Magliulo G., Maniaci A., Meccariello G., Cocuzza S., Cammaroto G., Gobbi R., Sgarzani R., Firinu E., Corso R.M. (2021). Olfactory function in patients with obstructive sleep apnea: A meta-analysis study. Eur. Arch. Oto-Rhino-Laryngol..

[24] Zou L.Q., Linden L., Cuevas M., Metasch M.L., Welge-Lüssen A., Hähner A., Hummel T. (2020). Self-reported mini olfactory questionnaire (Self-MOQ): A simple and useful measurement for the screening of olfactory dysfunction. Laryngoscope.

[25] Langstaff L., Pradhan N., Clark A., Boak D., Salam M., Hummel T., Philpott C.M. (2019). Validation of the olfactory disorders questionnaire for English-speaking patients with olfactory disorders. Clin. Otolaryngol..

